# Correction Notice: A CRISPR Competition Assay to Identify Cancer Genetic Dependencies

**DOI:** 10.21769/BioProtoc.3960

**Published:** 2021-02-20

**Authors:** Vishruth Girish, Jason M. Sheltzer

**Affiliations:** Cold Spring Harbor Laboratory, Cold Spring Harbor, NY 11724, USA

After official publication of our protocol in Bio-protocol (https://bio-protocol.org/e3682) we noted an error in the protocol.

In the Materials and Reagents section, the catalog number of VSVG plasmid is "98286", which is completely wrong. Correct catalog number of VSVG plasmid is "12259". Accordingly, we have now corrected as follows:

12. VSVG plasmid (Addgene, catalog number: 12259)

